# Determinants of psychological morbidity in survivors of the earthquake and tsunami in Aceh and Nias

**DOI:** 10.1186/1752-4458-4-8

**Published:** 2010-04-27

**Authors:** I Irmansyah, Suryo Dharmono, Albert Maramis, Harry Minas

**Affiliations:** 1 Department of Psychiatry, University of Indonesia, Kimia II No 35, Jakarta, 10430, Indonesia; 2 World Health Organization Indonesia, 9th Floor, Bina Mulia 1, Jalan Rasuna Said, Jakarta, Indonesia; 3 Centre for International Mental Health, Melbourne School of Population Health, University of Melbourne, Parkville, Victoria 3010, Australia

## Abstract

**Background:**

The goal of this study was to collect information to inform the design of a mental health response following the massive December 2004 earthquake and tsunami in Aceh and North Sumatra, Indonesia. As well as exploring the effect on mental health of direct exposure to the tsunami the study was designed to examine the effect on mental health of immediate post-disaster changes in life circumstances (impact).

**Methods:**

Information was collected from a sample of 783 people aged 15 years and over in earthquake and tsunami-affected areas of Aceh and Nias, 616 Internally Displaced Persons (IDPs) and 167 non-IDPs. The structured questionnaire that was designed for data collection consisted of demographic information, measures of disaster exposure and of changes in life circumstances (impact), the extended version of the Self-Reporting Questionnaire (SRQ), and a brief measure of resilience. Group comparisons, contrasting responses of IDPs and non-IDPs, were by chi-square for frequency data and t-tests for ordinal or continuous data. Hierarchical multiple linear regression analyses were performed to examine the relative contributions to psychopathology of demographic variables and measures of exposure, impact and resilience.

**Results:**

High rates of psychopathology, including symptoms of anxiety and affective disorders and post-traumatic stress syndrome, were recorded in the overall sample, particularly in Internally Displaced Persons (IDPs) who experienced more substantial post-disaster changes in life circumstances (impact). The IDP group experienced significantly more SRQ symptoms than did the non-IDP group. Demographic factors alone accounted for less two percent of variance in SRQ-scores. Higher SRQ-20 scores were observed among women, those with lower education, those with diminished resilience beliefs, those experiencing high scores on disaster impact, those experiencing direct exposures to the disaster, and due to (unmeasured) conditions related to being an IDP. The greatest effect among these was due to disaster impacts. The pattern was similar when considering post-traumatic stress symptoms separately.

**Conclusions:**

Negative changes in a person's life circumstances following a disaster appear to have as important an effect on psychopathology as the direct experience of the disaster. Ameliorating the extent and duration of post-disaster negative changes in life circumstances may play an important role in prevention of post-disaster psychological morbidity.

## Background

Norris' et al review [1,2] and update [3] of 225 samples that experienced major natural or technological disasters or were subject to mass violence indicated that the severity of mental and emotional disorder due to the traumatic event was appreciably higher in developing than developed countries. It was suggested that this might be due to disasters of greater magnitude occurring or being reported from developing countries or to the relative lack of supportive services to address the mental health needs of survivors. While it is clear from the majority of studies that direct exposure to traumatic events compromises mental health [1,2,4,5] particularly evident as higher prevalence of post-traumatic stress disorder, depression and anxiety disorders, how the post-disaster social and physical environment and changes in life circumstances may contribute to diminished mental health status remains an area for further research. Such knowledge is important to the development of comprehensive disaster responses that include addressing the mental health of survivors [4].

With the exception of work on social support, there has been little work to assess how post-trauma conditions may be related to psychopathology [1-5]. In a suggestive study, Fukuda et al [6] examined highly exposed Awaji Island survivors of the Hanshin-Awaji (Japan) earthquake at 20 months following the disaster. Those with more difficult life circumstances following the disaster, who were also more likely to be still living in temporary housing, had higher mean scores on a DSM-IV-based post-traumatic stress disorder measure. Those with more difficult life circumstances were 2.6 times more likely to be experiencing intrusive recollections and imagery, and 4.6 times more likely to have hyper-arousal symptoms. Unfortunately the study did not address the relative contribution of individual exposure and changes in life circumstances on psychopathology. Nevertheless, the general literature on the effects of stressful life events [7] would suggest an association between post-disaster adverse changes in life circumstances and increased morbidity. However, it is not clear, within a sample experiencing a major disaster, if this relationship is additive/cumulative between exposure and changes in life circumstances or the latter moderates the effects of exposure on morbidity. A moderation effect would suggest that exposure may increase the psychological impact of secondary post-disaster stressors. Further, work by Norris and associates [8,9] in survivors of hurricanes Hugo and Andrew suggests that post-disaster stressors may mediate between initial exposure and eventual psychopathology. That is, exposure predisposes those affected to secondary stressors, which in turn contribute to a decline in psychological health.

In the present work we explore the impact of post-disaster changes in life circumstances on psychopathology in addition to direct exposure experiences and other factors in the Indonesian communities of Aceh and Nias that experienced the 2004 Sumatra-Andaman earthquake and tsunami, with data collected at approximately two months following the event. Aceh is a province of Indonesia situated in the northern-most area of Sumatra. According to the 2001 Census, its population was 4.5 million, the majority concentrated in the flat fertile lands east of the Bukit Barisan ranges, and particularly in the costal towns and cities. The community is composed of several ethnic groups, Acehnese, Gayo, Tamiang, and Alas, there are four major dialects spoken and the majority is Muslim. To the time of the disaster the region had been embroiled in a protracted political and armed conflict between the Aceh Freedom Movement and the Central Government in Jakarta, with rising antipathy towards both sides in the local population due to the hostilities, victimisations and property damage. Martial Law was implemented in 2003, with 30,000 troops sent to suppress separatists, succeeded by Civil Emergency status, which ended in May 2005, following the disaster. Such socio-political conditions in developing countries are known to contribute to post-traumatic stress disorder [5,10].

On Sunday December 26th 2004 an earthquake of magnitude 9.1-9.3 on the Richter scale occurred off the west coast of Northern Sumatra [11], 255 kilometres from Banda Aceh. The earthquake, which produced peak to peak ground movement across the Earth's surface of 1 cm or more [11], caused extensive damage and casualties. The tsunami, arriving some 30 minutes later in Aceh, caused utter devastation. The tsunamis, spreading across the Indian Ocean, caused damage and casualties along the exposed coastlines of several other countries including, in particular, Sri Lanka, Thailand, and India. The worldwide death toll was estimated to be more than 280,000. The number of deaths in Aceh alone was 130,000 [12]. Mortality rates were highest in the young, children less than 10 years, and adults aged over 50 years [13].

In an effort to assess the situation and to inform the mental health response to the disaster, two rapid assessment population surveys were developed and conducted by the Department of Psychiatry, University of Indonesia, examining the prevalence of psychological disorder in adult and child samples within two months of the disaster. Here we report the findings from the adult sample, which was composed of two groups. The first consisted of persons displaced from their homes by the events (internally displaced persons, IDPs), the majority of whom were now living in temporary camps. These were considered a high exposure and high post-disaster negative changes in life circumstances impact group. The second group consisted of those residing on the periphery of the camps in their usual dwellings and, although not displaced and having lower direct exposure to the tsunami, they were not necessarily unaffected personally and socially by the disaster. An attempt was made to generally match the latter group to the first on age and gender.

In addition to examining the effects of exposure and post-disaster changes in life circumstances, the study included socio-demographic measures (age, sex, education) and a brief measure of resiliency beliefs [14]. The latter is thought of as a personality factor that may be protective against the development of psychopathology in the face of severe stressors [15,16]. Given that not all highly exposed individuals develop post-traumatic reactions, resilience has been suggested to moderate the relationship between degree of trauma exposure and diminished psychological health [15]. However other opinion holds that exposure to severe trauma actually shatters one's assumptions about the world and one's sense of control of events and life, suggesting that severe trauma exposure may in fact reduce personal invulnerability beliefs [17]. In this study we examine these relationships in models that take into account socio-demographic, exposure and post-disaster changes in life circumstances, and begin with an account documenting the psychological impact of the disaster on the survivors.

## Methods

### Sample

The study was carried out in areas affected by the December 2004 tsunami in Aceh and North Sumatra, Indonesia. The sample, people aged 15 years and above, consisted of two groups: Internally Displaced Persons (IDPs), who were affected directly by the disaster, and who were living in IDP camps, living temporarily in local people's homes, or were in hospital, and non-IDPs, people who were living in areas not directly affected by the tsunami and near camps where the IDPs were living.

A sample of 783 participated in the study. Of these, 616 were living in camps, in temporary housing or in hospital - the IDP group. The remaining 167 were permanent residents living close to the camps who had not been displaced from their homes by the disaster. The latter group was not necessarily un-exposed to the effects of the disaster but represented a group that was thought to have a lower degree of exposure than the IDPs. An attempt was made to select non-IDPs in the same age range as IDPs and equal numbers of males and females. These and other details of the groups are presented in the Results section (see Table 1). The sample was drawn from the following locations, Aceh Besar (n = 148, 18.9%), Banda Aceh (n = 152, 19.4%), Calang (n = 96, 12.3%), Lokseumawe (n = 128, 16.3%), Meulaboh (n = 132, 16.9%), and the island of Nias (n = 127, 16.2%).

**Table 1:** Comparative characteristics of IDPs and non-IDPs and total sample descriptive statistics

	IDP group N = 616	non-IDP group n = 167	Statistical Outcome	Total Sample N = 783
**Demographics**				
Mean Age (sd)	34.5 (13.5)	33.2 (12.7)	ns^1^	34.2 (13.4)
Sex (% male)	46.3	50.9	ns^2^	47.3
Mean Education (sd)	1.86 (1.21)	2.75 (1.31)	***^3^	2.05 (1.28)
Muslim (%)	88.0	92.2	ns^5^	88.9

**Living in (% in)**			***^4^	
House	23.9	100		40.1
Camp	72.1	0		56.7
Hospital	4.1	0		3.2

**Tsunami exposure (% yes)**				
In disaster zone/saw wave	93.2	6.0	***^6^	74.6
Experienced wave/earthquake	84.9	34.7	***^7^	74.2
Physically injured	33.3	1.2	***^8^	26.4
Death immediate family	40.9	17.4	***^9^	35.9
Missing immediate family	32.0	16.2	***^10^	28.6
Death extended family	68.0	61.1	ns^11^	65.5
Missing extended family	55.0	58.7	ns^12^	55.8
Property loss	92.5	18.6	***^13^	76.8
Swept by wave/almost killed	39.4	0.0	***^14^	31.0
Mean sum exposures (sd)	5.39 (1.94)	2.13 (1.47)	***^15^	4.70 (2.28)

**Tsunami impact (% yes)**				
On job	54.2	32.9	***^16^	49.7
Household activities	40.9	23.9	***^17^	37.3
Friendships	31.9	24.4	ns^18^	30.3
Recreation	44.2	40.9	ns^19^	43.5
School/study	13.6	14.0	ns^20^	13.7
On family relationships	25.3	15.2	**^21^	23.2
Sexual activity	27.1	8.5	***^22^	23.2
Life in general	52.7	39.6	**^23^	49.9
Daily activities	41.7	41.5	ns^24^	41.7
Mean sum impact (sd)	3.28 (2.34)	2.37 (2.22)	***^25^	3.08 (2.35)

**Resilience beliefs**				
Psychological	2.29 (1.22)	2.31 (1.22)	ns^26^	2.30 (1.22)
Physical	2.38 (1.31)	2.41 (1.28)	ns^27^	2.39 (1.30)

**Psychopathology**				
Mean total SRQ-20	10.32 (4.51)	6.43 (4.69)	***^28^	9.49 (4.91)
Mean total post-traumatic symptoms	2.24 (1.62)	1.50 (1.53)	***^29^	2.08 (1.63)
Increased alcohol use (%yes)	3.2	1.8	ns^30^	2.9
Someone will harm one (%yes)	7.5	3.6	ns^31^	6.6
Interference in thoughts (%yes)	24.8	13.8	**^32^	22.5
Hearing voices (%yes)	26.5	12.0	***^33^	23.4

^1 ^t(781) = 1.12, p = .263

^2 ^c^2^(1) = 1.13, p = .288

^3 ^t(781) = 8.25, p < .001

^4 ^c^2^(2) = 317.06, p < .001

^5 ^c^2^(1) = 2.38, p = .123

^6 ^c^2^(1) = 526.94, p < .001

^7 ^c^2^(1) = 172.77, p < .001

^8 ^c^2^(1) = 69.44, p < .001

^9 ^c^2^(1) = 31. 65, p < .001

^10^c^2^(1) = 16.09, p < .001

^11 ^c^2^(1) = 2.84, p = .092

^12 ^c^2^(1) = .71, p = .400

^13 ^c^2^(1) = 402.92, p < .001

^14 ^c^2^(1) = 95.52, p < .001

^15 ^c^2^(1) = 23.36, p < .001

^16 ^t(339.5) = 23.61, p < .001

(unequal variances assumed)

^17 ^c^2^(1) = 16.17, p < .001

^18 ^c^2^(1) = 3.41, p = .065

^19 ^c^2^(1) = .579, p = .447

^20 ^c^2^(1) = .017, p = .896

^21 ^c^2^(1) = 7.32, p < .01

^22 ^c^2^(1) = 25.00, p < .001

^23 ^c^2^(1) = 8.84, p < .01

^24 ^c^2^(1) = .003, p = .955

^25 ^t(781) = 4.50, p < .001

^26 ^t(777) = .177, p = .859

^27 ^t(777) = 4.50, p = .797

^28 ^t(781) = 9.57, p < .001

^29 ^t(781) = 5.25, p < .001

^30 ^c^2^(1) = .97, p = .325

^31 ^c^2^(1) = 3.18, p = .074

^32 ^c^2^(1) = 9.23, p < .01

^33 ^c^2^(1) = 15.39, p < .001

### Procedure

All camps or other temporary housing in each relevant local area were identified. Six camps were randomly chosen. The lists of IDPs living in each camp were the population from which study participants were recruited using a stratified random sampling method. Six research teams carried out the sampling and data collection. The study was conducted from February 21 to March 7 2005, 6 to 8 weeks after the tsunami disaster.

At least four local data collectors were recruited in each site. The data collectors received a one-day training and familiarization program carried out in the local area. Where research participants did not speak the national language (Bahasa Indonesia) the data collectors were able to conduct the research interview in the relevant local language. All interviews took place in the IDP camps, in people's homes or in hospitals.

Participants were approached, given information about the purpose and procedure of the research, and invited to participate. Only those who gave informed consent were interviewed. Participants who displayed significant distress or disorder were offered counselling or psychiatric treatment as necessary and referred to appropriate services. Participants were preferentially referred to local medical services that existed prior to the disaster to ensure continuing access to services when required.

Permission to carry out the study was given by the Ministry of Health, Republic of Indonesia.

### Instruments

A structured questionnaire was used to interview participants, comprising of:

a. brief demographic section (measuring age, sex, education, religion),

b. a list of nine exposure measures relating to the earthquake and tsunami (including such items as seeing the wave, being swept by the wave and near death, being injured, death or disappearance of family members, property loss),

c. nine impact measures (including such items as impact on job, school, household activities, friendships and family relationships),

d. an extended version [18] of the Self-Reporting Questionnaire (SRQ) with additional items covering use of alcohol (one item), symptoms of psychosis (three items) and symptoms of post-traumatic stress disorder (five items). All responses on exposures, impacts and symptoms were binary (answered yes or no). Since part of the analysis to follow examines exposures and impacts as scales, reliability coefficients were calculated for these. In relation to exposures the alpha coefficient for the sample was .72 (IDP alpha = .63; non-IDP alpha = .50). For impacts the overall alpha coefficient was .73 (IDP alpha = .73; non-IDP alpha = .75),

e. two questions (the Connor-Davidson Resilience Scale - 2) gauging sense of personal resilience in relation to psychosocial stressors and physical injury, illness or hardship. For convenience these will be referred to as Psychological Resilience and Physical Resilience. The questions were answered on a five-point scale ranging from 'not true at all' to 'true nearly all the time'. When combined the alpha coefficient was .64 (IDP alpha = .62; non-IDP alpha = .72). And,

f. Other items, not subject to the present analysis, asked about speed of and satisfaction with the timeliness of receiving support from authorities and type of temporary housing currently or previously used.

The SRQ was developed by the World Health Organization to be applicable across different cultures and settings. The User's Guide [19] indicates that the most common use of the SRQ-20 is to measure psychological distress, including symptoms of anxiety and dysphoric states. An additional three items included in the Ehrenreich and McQuaide extended version of the SRQ [18] capture psychotic experiences (thoughts that others are trying to harm the person, awareness of interference or anything unusual in one's thinking, and hearing voices others cannot hear). However, these were not recommended by the User's Guide due possible cultural misunderstanding and the influence of lack of insight when the person is suffering psychosis. In the present analysis these items are considered exploratory and analysed separately. One additional item measures increased use of alcohol during the period of measurement and this too is treated separately in the analysis. The five items to measure symptoms of post-traumatic stress disorder suggested by Ehrenreich and McQuaide [18] included vivid distressing dreams about the disaster, avoidance of reminders, loss of interest in activities, distress when reminded about the disaster and numbing of emotional experience or expression. The alpha coefficient of the SRQ-20 in the present sample was .85 (and did not differ according to group: IDP alpha = .84; non-IDP alpha = .85) and for the post-traumatic symptoms scale .68 (IDP alpha = .67; non-IDP alpha = .69).

All instruments were translated and adapted for use in Indonesia. Pre-tests of the instruments, to examine feasibility and acceptability, were carried out in Jakarta in a convenience sample of fifty healthy volunteers and thirty adult psychiatric patients.

### Design and Analysis

There were two groups, hypothesised to have different levels of exposure and impact due to the disaster - IDPs and non-IDPs. Simple descriptive statistics were calculated to examine the total sample and group characteristics on demographic, exposure, impact, resilience and psychopathology variables. Group comparisons, contrasting responses of IDPs and non-IDPs, were by chi-square for frequency data and t-tests for ordinal or continuous data.

The relative contributions of impacts, exposures, resilience as well as demographic variables on psychopathology were examined through the use of hierarchical multiple linear regression analyses. All binary responses were dummy coded as one (no/males) and two (yes/females). The education variable was dummy-coded numerically ranging from 1, incomplete primary school, to six, completed tertiary education. Demographic variables were entered first to examine the effects of other variables once these are held constant. Order of entry of the demographic factors was from that with the lowest to that with the highest zero order correlation with the psychopathology dependent measure. Next resilience scores were entered, considered as a possible personality factor that may be controlled prior to exploring the effects of impacts and exposures. Impact scores were then entered on the basis of their higher zero order correlation with the psychopathology measures followed by exposure scores to explore if exposures added any further prediction beyond impact scores. Lastly IDP group status was entered to examine whether any unmeasured factors related to IDP group membership improved the model's prediction over and above all other factors. To test moderator effects (exposure × impact, exposure × resilience, and, impact × resilience) interaction terms were calculated by Burrill's partial orthogonalisation method [20] and checked against the centering method (both resulted in the same outcomes).

## Results

### Univariate description of the sample and groups

Descriptive statistics are presented in Table 1. The mean age was 34 years and approximately half of the sample was male. Groups did not differ in these characteristics, consistent with the sampling strategy. However non-IDPs had significantly higher education level than IDPs with the latter averaging between completion of primary school and junior high school and the former between junior high school and senior high school. As indicated in Table 1, IDPs were mostly living in camps at the time of the study while others temporarily in houses, and a small group in hospital, having received injuries from the disaster. The majority in both groups were Muslim with approximately similar proportions.

In relation to direct exposures to the disaster it is evident from Table 1 that the non-IDPs were also affected in large numbers. While physical injury and direct experience of the tsunami wave were low in this group they too were substantially exposed to deaths and missing family members, and particularly members of their extended family, experience of the earthquake and property loss. Apart from death of, and missing, extended family members, significantly greater proportions of the IDP group experienced a wide range of direct disaster exposures, ranging from 32 percent experiencing missing members of their immediate family through to 93 percent reporting loss of valuable possessions and property. Overall the IDP group experienced on average five of the nine listed exposures compared with two for the non-IDP group.

The same general pattern pertains to impacts of the disaster. Depending on the nature of the impact, between 14 and 50 percent of the overall sample reported an impact. Impacts that did not differ in frequency between groups included those on friendships, recreation, school/study and on daily activities. Significantly higher proportions of IDPs reported impacts on work, household activities, family relationships, sexual activity and life in general. Overall IDPs reported experiencing over three impacts compared with over two impacts for the non-IDP group and this difference was significant (Table 1).

Sense of resilience did not differ between groups for either psychological or physical resilience. The mean scores on these items suggest ratings of resilience between 'sometimes true' to 'often true' with a bias towards the lower value.

Mean SRQ-20 scores for the overall sample indicate that the sample reported having experienced nine to ten symptoms, and significantly more symptoms were reported by the IDP group (ten) than the non-IDP group (six). The IDP group also reported significantly more post-traumatic symptoms than the non-IDP group, the former group averaging over two symptoms compared to the latter averaging between one and two (of the five). Increased use of alcohol was low in prevalence with only three percent reporting this and no difference was observed between IDP and non-IDP groups on this. In regards to the putative symptoms of psychosis, while 'thoughts that others were setting out to harm the person' was low in prevalence (seven percent) a surprisingly high proportion of the sample (approximately 23 percent) reported 'thought interference' and 'hearing voices'. In view of any validation of cut-off scores for 'neurosis' according to the SRQ-20 in an Indonesian sample, several criteria were calculated comparing IDP and non-IDP groups. The most common cut-off used in different national samples is 7/8 and for this close to 71 percent of the IDP group met such criteria compared with 36 percent of the non-IDP group. Case rates for the IDP group did not decline appreciably until the 12/13 criterion was applied. In relation to post-traumatic symptoms scale, according to Ehrenreich and McQuaide [18], a score of one or higher warrants clinical follow-up. This cut-off suggests an extremely high rate of possible cases, regardless of group membership, with 81 and 63 percent of the IDP and non-IDP groups meeting this criterion (77.1 percent overall). Even if three (of five) symptoms is used as the criterion, over 40 percent of the sample would possibly classify as having post-traumatic syndrome (46 percent for IDPs and 25 percent for non-IDPs).

When analysis focused on specific symptoms from the SRQ-20 and post-traumatic stress questionnaire, significantly greater proportions of IDPs than non-IDPs endorsed every symptom. Among the most common symptoms (occurring in over 60 percent of the overall sample) were headaches, nervousness, fright, and sleep disturbances. Symptoms where there was 20 percent or higher difference in prevalence in the IDP than non-IDP group were: tiredness (30%), crying (28.5%), work/study difficulties (26.9%), unhappiness (25.6%), digestive problems (21.5%), lack of enjoyment (20.8%), hands shaking (20.4%) and difficulty in decisions (20.0%). Of concern, thoughts of ending one's life were reported by 11 percent of the IDP group and 3.6 percent by the non-IDP group.

### Modelling psychological distress and post-traumatic symptoms

Hierarchical multiple linear regression analyses were conducted to examine the contributions of demographic, impact, exposure, and resilience variables on psychopathology. Results are summarised in Table 2 for the prediction of SRQ-20 scores (upper half of Table 2) and total post-traumatic symptoms (lower half of Table 2). In Table 2, zero order correlations and beta coefficients are presented in a matrix format detailing the effects of successive variable entries into the model - giving insight into the relative independence of the factors in predicting psychopathology and highlighting possible direct and mediated effects [21].

**Table 2:** Hierarchical multiple linear regressions predicting SRQ-20 scores and post-traumatic symptoms total scores

Factor	Zero Order r	Model 1	Model 2	Model 3	Model 4	Model 5	Model 6	Model 7
*SRQ-20 scores*								
Age	.003^ns^	.003^ns^	.016^ns^	-.030^ns^	-.027^ns^	-.011^ns^	-.020^ns^	-.012^ns^
Sex	.137***	-	.138***	.122**	.087*	.083**	.083**	.082**
Education	-.160***	-	-	-.158***	-.161***	-.169***	-.164***	-.122***
Resilience	-.299***	-	-	-	-.289***	-.194***	-.175***	-.187***
Impact	.416***	-	-	-	-	.366***	.319***	.310***
Exposure	.334***	-	-	-	-	-	.238***	.154***
IDP grouping	.324***	-	-	-	-	-	-	.146***

Change in percent variance	-	0	1.9	2.3	8.2	12.4	5.4	1.2
F change	-	<1	14.81***	18.28***	71.57***	126.18***	58.43***	13.58***

Model F	-	<1	7.41**	11.15***	27.02***	50.40***	54.90***	49.77***
Adjusted R^2^	-	.000	.016	.038	.119	.243	.296	.308

*Post-Traumatic symptoms*								

Education	-.057^ns^	-.057^ns^	-.046^ns^	-.093*	-.095**	-.103**	-.099**	-.105**
Sex	.143***	-	.139***	.121***	.094**	.089**	.089**	.090**
Age	-.150***	-	-	-.164***	-.162***	-.147***	-.154***	-.155***
Resilience	-.239***	-	-	-	-.118***	-.128***	-.112***	-.111***
Impact	.412***	-	-	-	-	.370***	.330***	.331***
Exposure	.287***	-	-	-	-	-	.203***	.215***
IDP grouping	.185***	-	-	-	-	-	-	-.021^ns^

Change in percent variance	-	0.32	1.9	2.5	5.0	12.7	3.9	0.0
F change	-	2.64	15.08***	19.76***	41.93***	124.76***	40.13***	<1

Model F	-	2.46^ns^	8.79***	12.59***	20.43***	43.94***	45.19***	38.73***
Adjusted R^2^	-	.002	.020	.043	.092	.219	.257	.256

ns = not significant

* p < .05

** p < .01

*** p < .001

Beta coefficients appear in the upper diagonal matrices. Coefficients in bold type depict values with all preceding variables entered in the model. Zero order correlations (r) are shown for comparing change in beta values across models. Effects of factor entry and model statistics appear below each matrix.

As indicated in Table 2, demographic factors alone accounted for less two percent of SRQ-scores variation. Age was not a predictor in any of the SRQ-20 models. The effect of gender was positive and education negative, indicating that women scored higher than men and that higher psychopathology was reported among those with lower education. Introduction of education (Model 3) and resilience (Model 4) lowered the beta coefficient of gender suggesting that some of the effect of gender on SRQ-scores was mediated by the lower education and lower perceived resilience in women relative to men. In general, resilience appears to have an effect on SRQ-20 scores independent of demographic factors as indicated by the lack of change in the beta coefficient in the presence of the demographic predictors (Model 4). Entering impacts into the model (Model 5) led to a large increase in the variance accounted for by the model. The beta coefficient for resilience diminished in size possibly reflecting the effect of impacts on lowering resilience. Importantly, the direct effect remained significant. In Model 6, where disaster exposure is entered, its own direct effect is reduced (but remained significant) by the presence of the other variables in the model and particularly resilience and impacts. Model 7 shows the effect of entering IDP group membership, which diminishes the beta coefficients of exposure and impacts suggesting the IDP-psychopathology pathway is partly through these factors. Regardless of the variations in beta coefficients across the models, indicative of mediated effects, the final model (Model 7) suggests direct and independent effects on SRQ-20 scores from all predictors except age. The model accounted for 31 percent of the variance based on the Adjusted R^2 ^estimate. In sum, higher SRQ-20 scores were from women, those with lower education, those with diminished resilience beliefs (in part a function of exposures and particularly impacts), those experiencing high scores on disaster impacts, those experiencing direct exposure to the disaster and due to (unmeasured) conditions related to being an IDP. The greatest effect among these was due to disaster impacts.

Turning to the findings relating to post-traumatic symptoms, the best model was Model 6 accounting for 26 percent of the variance (Table 2). In relation to the demographic predictors entering additional variables, particularly age, into the equation (Model 3) uncovered a suppressor effect [22]. As above, several indirect effects are suggested, and in the case of the effect of IDP group membership on morbidity scores, this appears to be fully accounted for by impact and exposure scores. But the most important aspect of the findings remains the direct independent contributions to the variation in traumatic symptom scores attributable to the predictors. These were, lower education, female gender, younger age, lower resilience scores, higher impacts and higher exposures. As in the case of SRQ-20 scores, the strongest predictor of post-traumatic symptoms was disaster impact score.

Last we examined the moderation effects taking a two-factor model in each case. With respect to SRQ-20 scores, a significant interaction term was observed. While each of the primary variables, exposure and impact, were positively related to higher SRQ-20 scores the interaction had a small additional negative relationship to these (Beta = -.065, t = 2.065, p = .039), adding only 0.4 percent to the variance explained by the main factors. We interpret this to mean a negligible influence on scores which increase predominantly in association with exposure (Beta = .251, t = 7.78, p < .0005) and impacts (Beta = .362, t = 11.19, p < .0005) (F _main effects model _(2, 770) = 116.94, p < .0005, Adjusted R^2 ^= .23). No other test of moderation (exposure × resilience, impact × resilience) proved significant in relation to SRQ-20 scores and none of the three tests were significant in relation to traumatic symptom scores.

It is noteworthy, however, that while the findings show that disaster-related phenomena are associated with psychopathology, as much as 75 percent of the variance in psychopathology remains unexplained in this sample.

## Discussion

The adult communities of Aceh and Nias, assessed at two months following the disaster, suffered high levels of psychological morbidity. High rates of psychopathology, including symptoms of anxiety, affective disorders and post-traumatic stress syndrome, were recorded in the overall sample, particularly in those most directly affected by the tsunami. This group, IDPs, was also characterised by more substantial post-disaster changes in life circumstances in addition to their direct exposures to the tsunami. Results in relation to the effects of exposure are consistent with most other studies of tsunami survivors in Aceh and disaster survivors elsewhere [1,2,23-25].

Less clear has been the role of post-disaster changes in life circumstances on psychopathology. The present work suggests that the post-disaster changes in life circumstances, at least in this sample, are directly related to psychopathology, irrespective of direct exposure to the disaster. Indeed all the key factors studied - exposure, resilience and impacts - had direct effects on psychopathology, even after controlling for IDP status. Further, while there was little evidence to suggest moderated relationships between exposure, resilience and impacts, several results point to additional mediation effects. For example, exposures and impacts appear to be associated with reduced resilience, and together with direct effects from the primary factors influence variation in psychopathology (whether measured by the SRQ-20 scores or by the number of endorsed post-traumatic stress disorder symptoms). Given the temporal contiguity of exposures and impacts it would appear that impacts in part mediate the relationship between exposure and morbidity, as suggested by previous research [23,26,27]. The significant role of disaster impacts, including reduced sense of resilience, implies an important role for morbidity prevention programs for those with direct or indirect exposures to the disaster. In the post-disaster period, together with improvements in the conditions of living of survivors there is a need to address the psychological meaning of the disaster and its aftermath, including the emotional effects of personal, social and property losses, the role of physical dislocation, the confusion and disruption of routine life, and the effects of fostering hope and self-efficacy.

A significant limitation in the study design was the lack of information on exposures to the pre-disaster political conflict so that the relative influence on psychopathology of pre-disaster conditions cannot be examined. The measured variables - exposure, resilience and impacts, and to some extent education and gender - explain almost one third of observed variance in psychological morbidity and only a quarter of observed variance in post-traumatic symptoms. Approximately three quarters to two thirds of variance in post-traumatic symptoms and psychological morbidity is not explained by the measured factors. Other factors that may increase vulnerability or that may confer protection against development of psychopathology (e.g. social support), and pre-existing morbidity that may be related to pre-disaster social, political and economic conditions, need to be directly examined in order to enable more complete examination of the effects of disaster exposure and post-disaster conditions.

Lessons have been learned from the 2004 disaster, including better disaster preparedness and the need for a quick and appropriate mental health and psychosocial response [28,29] and the disaster in Aceh has created the opportunity and impetus to develop new models of mental health care with a community focus [30].

## Conclusions

A high proportion of the residents of Aceh and Nias experienced psychological symptoms after the disaster, with IDPs being more severely affected than non-IDPs. Direct traumatic experience of being exposed to the tsunami and the impact of the disaster on circumstances of life after the disaster had independent impact on probability of developing psychological symptoms. Ameliorating the extent and duration of post-disaster negative changes in life circumstances may play an important role in prevention of post-disaster psychological morbidity.

## Competing interests

The authors declare that they have no competing interests.

## Authors' contributions

Ir, SD and AM were primarily responsible for the study design and management of data collection, did the field work and organized the research teams. Ir did the primary data analysis and report. HM provided technical input during the study design phase. Data analyses were performed by SK (see Acknowledgements) and SK, Ir and HM were responsible for interpretation of study findings and writing of the first draft of the manuscript. HM wrote the final draft of the manuscript, which has been reviewed and approved by all authors.
